# Identification of prognostic subtypes and the role of FXYD6 in ovarian cancer through multi-omics clustering

**DOI:** 10.3389/fimmu.2025.1556715

**Published:** 2025-03-18

**Authors:** Boyi Ma, Chenlu Ren, Yun Gong, Jia Xi, Yuan Shi, Shuhua Zhao, Yadong Yin, Hong Yang

**Affiliations:** Department of Obstetrics and Gynecology, Xijing Hospital, The Fourth Military Medical University, Shaanxi, China

**Keywords:** ovarian cancer, multi-omics, machine learning, FXYD6, prognostic

## Abstract

**Background:**

Ovarian cancer (OC), as a malignant tumor that seriously endangers the lives and health of women, is renowned for its complex tumor heterogeneity. Multi-omics analysis, as an effective method for distinguishing tumor heterogeneity, can more accurately differentiate the prognostic subtypes with differences among patients with OC. The aim of this study is to explore the prognostic subtypes of OC and analyze the molecular characteristics among the different subtypes.

**Methods:**

We utilized 10 clustering algorithms to analyze the multi-omics data of OC patients from The Cancer Genome Atlas (TCGA). After that, we integrated them with ten different machine-learning methods in order to determine high-resolution molecular subgroups and generate machine-learning-driven characteristics that are both resilient and consensus-based. Following the application of multi-omics clustering, we were able to identify two cancer subtypes (CSs) that were associated with the prognosis. Among these, CS2 demonstrated the most positive predictive outcome. Subsequently, five genes that constitute the machine learning (ML)-driven features were screened out by ML algorithms, and these genes possess a powerful predictive ability for prognosis. Subsequently, the function of FXYD Domain-Containing Ion Transport Regulator 6 (FXYD6) in OC was analyzed through gene knockdown and overexpression, and the mechanism by which it affects the functions of OC was explored.

**Results:**

Through multi-omics analysis, we ascertained that the high-risk score group exhibits a poorer prognosis and lack of response to immunotherapy. Moreover, this group is more prone to display the “cold tumor” phenotype, with a lower likelihood of benefiting from immunotherapy. FXYD6, being a crucial differential molecule between subtypes, exerts a tumor-promoting effect when knocked down; conversely, its overexpression yields an opposite outcome. Additionally, we discovered that the overexpression of FXYD6 can induce ferroptosis in OC cells, implying that a low level of FXYD6 in OC cells can safeguard them from ferroptosis. Insightful and more precise molecular categorization of OC can be achieved with a thorough examination of multi-omics data. There are significant consequences for clinical practice stemming from the discovery of risk scores since they provide a useful tool for early prognosis prediction as well as the screening of candidates for immunotherapy.

## 1 Introduction

From a global perspective, ovarian cancer (OC) is the leading cause of cancer-related deaths among women and the eighth most prevalent malignancy overall among all cancers in women (1, 2). Endometrial cancer (3), chronic myeloid leukemia (4), ER (+) or Her2 (+) breast cancer (5), and EGFR-mutated lung cancer (6) have all reported successful use of molecularly targeted therapies based on the molecular classification of malignant tumors, owing to the ongoing advancements in cutting-edge technologies including genomics as well as proteomics. Currently, the genetic mechanisms of OC have been widely validated. Pathogenic allele mutations in BRCA1 and BRCA2, homologous recombination genes, and Lynch syndrome have been applied in clinical diagnosis (7, 8).

Prior research has put forth a lot of effort to identify the molecular subtypes of OC in order to ascertain the heterogeneity. A particular instance is the work of Tothill et al. (2008), who used K-means clustering to classify miRNA gene expression profiling microarray experiments into six distinct subtypes (9). Tan et al. used functional genomics to classify five molecular subgroups (10). Kommoss et al. assessed the correlation between TCGA molecular subtypes and the effectiveness of bevacizumab in a random assignment study, which has important implications for clinical practice. Median progression-free survival (PFS) improved for proliferative and mesenchymal molecular subtypes, which had the lowest survival rates, but overall survival (OS) did not alter significantly (11). However, our understanding of the molecular characteristics and subtypes of OC still requires further exploration. There is still room for molecular subtypes to enable more accurate diagnosis, treatment, and survival prediction. Therefore, starting from clinical practice, we aim to identify the molecular subtypes most relevant to prognosis through multi-omics analysis to distinguish patients and construct a stable classification selector.

With the use of ten multi-omics integration techniques, we were able to construct exhaustive consensus subtypes of OC by integrating genomic alterations with mRNA, long non-coding RNA (lncRNA), and microRNA (miRNA) expression patterns. We next used ten ML methods to generate consensus molecular subtypes and, based on the differentially expressed genes (DEGs) among the subtypes, determined five stable genes related to prognosis. The molecular subtypes showed strong predictive power for immunotherapy and medication-related outcomes in both the validation and training cohorts, and they were also a significant indicator of prognosis. Our study’s results are a vital benchmark for improving the accuracy of OC molecular subtypes, improving the categorization of cancerous tumors, and developing more tailored treatment strategies.

## 2 Methods

### 2.1 Data preprocessing of ovarian cancer multi-omics and multi-center cohort data

Initial data was collected from the OC cohort in The TCGA (https://portal.gdc.cancer.gov), which included patients with full transcriptome expression, somatic mutations, and accessible clinical information, as well as multi-omics data. The TCGAbiolinks package was used to derive the transcriptome profiles of mRNA and lncRNA (12). Using the miRBaseVersions.db package, the TCGA mature miRNAs’ IDs were documented. Additionally, somatic mutations were obtained via TCGAbiolinks and analyzed using the maftools program. From the Gene Expression Omnibus (GEO) database, we also retrieved the corresponding clinical data and two expression profiles (GSE26193, GSE49997) pertaining to OC. All expression profiles acquired using arrays were duplicated and normalized, and the transcriptome’s high-throughput sequencing was transformed into transcripts per million (TPM) using kilobase.

The expression matrices and clinical information used in the microarray data were obtained from the official websites. The data was processed using the robust limma software after being downloaded. This package performed background correction, log_2_ transformation, and quantile normalization. To visualize the data and examine the uniformity of the distribution of sample expression abundance values, the boxplot function was employed. If more than one probe was associated with a given gene symbol, the one with the greatest expression level was used to annotate the gene. We utilized TPM values for RNA-seq data from high-throughput sequencing because they improve sample comparability and are more equivalent to microarray gene expression (13). For the merging of different datasets, the “ComBat” function in the sva package was employed to adjust the batch effects of non-biotechnological biases for each dataset using an empirical Bayes framework (14). Many high-quality studies have reported similar algorithms, ensuring the scientific validity of our approach (15–17). To further verify the effectiveness of data merging, we also performed principal component analysis (PCA) on the merged samples before and after the merging process (18).

### 2.2 Multi-omics consensus integration analysis

The first step that we performed in order to properly carry out a complete analysis was to match the omics information of four dimensions by sample ID. In the end, the training set consisted of 276 samples that had all of their dimensions complete. A logarithmic transformation was performed on the TPM expression data. In the gene mutation matrix, we discovered mutations whenever genes featured any of the following non-synonymous variations: frameshift insertions or deletions, in-frame insertions or deletions, nonsense or missense or non-stop mutations, or splice site or translation start site mutations. These mutations were found in genes that contained any of the aforementioned variations. For this investigation, we screened gene signatures using the “getElites” function of the MOVICS package, which stands for Multi-Omics Integration VIsualization in Cancer Subtypes. We screen the top 1,500 genes with the largest degree of variation by setting the “method” parameter of the “getElites” function to “mad” for continuous variables (mRNA, lncRNA, miRNA). Afterwards, we tied clinical data with the “method” parameter set to “Cox” in order to find the predictive genes with a p-value less than 0.05 in every data dimension. Prior to analyzing the gene mutation data for binary variables, we screened the top 5,000 genes with the highest mutation degree using the “oncoPrint” function of the maftools package. Next, we reset the “method” option to “freq” in order to find the five most commonly mutated genes, which allowed us to perform additional screening based on mutation frequency. We included the results from these four aspects in our investigation so we could analyze them more extensively. We continued to identify the best number of clusters for our investigation after the initial feature selection. It is commonly understood that the ideal data cluster size is between small enough to minimize noise and large enough to keep relevant information. Hence, we utilized the “getClustNum” method from the MOVICS package to determine the number of subgroups. This method incorporates the Clustering Prediction Index (CPI), the gap statistic, and the Silhouette score. Our comprehension of OC from clinical practice and the recently conducted research led us to classify the OC omics subtypes into two groups. After that, we conducted a clustering analysis using the “getMOIC” tool. We employed the standard settings specified by the MOVICS software and entered ten clustering algorithms as the “methodslist” parameter: CIMLR, ConsensusClustering, SNF, iClusterBayes, PINSPlus, moCluster, NEMO, IntNMF, COCA, and LRA. The outcomes of each method’s grouping were subsequently obtained. After the 10 techniques’ clustering results were computed, we improved the clustering’s robustness by integrating the results of several algorithms employing the “getConsensusMOIC” function, which relies on the notion of consensus clustering. Both the “distance” and “linkage” parameters were set to “Euclidean” and “average” respectively (19). Eventually, our clustering results were obtained.

### 2.3 Subtype profiling and immune landscape analysis

Gene Set Variation Analysis (GSVA) was used to determine the enrichment scores of characteristics linked to multi-therapy (20). A total of 71 potential regulators linked to malignant chromatin remodeling and 23 transcription factors (TFs) pertaining to induced/suppressed targets were utilized in the construction of the transcriptional regulatory networks (TRNs) (regullons) that were made possible by the Reconstruction of TRNs and Regulators Analysis (RTN) R package. Our next step was to use the ESTIMATE R program to estimate the immune/stroma scores of tumor tissues and evaluate the distribution of immunological checkpoints among these subgroups. It was also determined by GSVA whether or not 24 different kinds of tumor immune microenvironment cells were enriched. Before comparing the consensus clustering to the Nearest Template Prediction (NTP) and Prediction Analysis of Microarrays (PAM) classifiers, in order to ensure the subtype stability, we validated the clustering findings with biomarkers specific to each subtype in the cohort that was used for testing (19).

### 2.4 Establishing a consensus prognostic profile driven by machine learning

We combined ten various approaches to ML in order to create a molecular categorization that has improved precision and a capacity to generalize. These algorithms are as follows: CoxBoost, Stepwise Cox, Lasso, Ridge, Elastic Net (Enet), Survival Support Vector Machines (survival-SVMs), Generalized Boosted Regression Models (GBMs), Supervised Principal Components (SuperPC), Partial Least Cox (plsRcox), and Random Survival Forests (RSF). Selecting features is a capacity that is incorporated into algorithms such as Stepwise Cox, RSF, CoxBoost, as well as Lasso. In the model construction phase, we utilized the TCGA cohort as the training set for the initial model development. Initially, we invoked the “optimCoxBoostPenalty” function to ascertain the optimal penalty (shrinkage) value for the CoxBoost model. Subsequently, we integrated it with cross-validation and executed 10-fold cross-validation on the CoxBoost model to identify the best number of boosting iterations. The “CoxBoost” function was ultimately employed to fit the model. A stepwise Cox analysis was conducted utilizing the survival package, and the intricacy of the statistical model was assessed employing the Akaike Information Criterion (AIC). The entire range of potential direction parameter combinations, encompassing “both”, “backward”, and “forward”, was computed. Utilizing the “cv.glmnet” function and the glmnet package, the Lasso, Ridge, as well as Enet models were implemented. We used 10-fold cross-validation to get the regularization value lambda and an interval of 0.1 for the trade-off parameter alpha. Execution of Ridge occurs when alpha is equal to 0, whereas execution of Lasso occurs when alpha = 1. In all other cases, Enet will be run. The “survivalsvm” function in the survivalsvm package was used to perform support vector analysis on datasets containing survival consequences, which allowed for the implementation of the survival-SVM model. Employing the gbm package, the GBM model was implemented. Fitting the GBM was done using 10-fold cross-validation with the “gbm” function. The superpc package, an expansion of PCA, was used to implement the SuperPC model. To conduct 10-fold cross-validation, the “superpc.cv” function was also employed. The plsRcox package’s “cv.plsRcox” function was utilized for the plsRcox model. Two crucial parameters, “ntree” and “nodesize,” were passed into the “rfsrc” function within the randomForestSRC package in order to generate the RSF model. For a random forest, “ntree” is the number of trees, and “nodesize” is the smallest size for the terminal nodes. For this investigation, we decided to use “ntree” set to 1,000 and “nodesize” set to 5 (21).

The molecular classification was developed and constructed in the following manner: analysis was performed in both the TCGA-OC and GEO cohorts. In all cohorts, genes exhibiting a p-value below 0.05 and a consistent orientation of hazard ratio (HR) were classified as stable prognosis-related genes. Ten machine-learning algorithms were employed. Ninety-five algorithm combinations were employed to develop the most predictive molecular classification, achieving excellent concordance index (C-index) performance. Subsequent to developing the models on the training set, we conducted further testing on the validation cohort. We computed the average C-index for each model, identifying the model with the greatest value as the best one.

### 2.5 Prognostic value and clinical application prospect of molecular subtypes

We evaluated every sample in the training as well as validation sets with respect to the resultant models and categorized the samples into high-risk (HRG) and low-risk (LRG) groups according to their results. The prognostic importance of the risk ratings was assessed using Kaplan-Meier survival curves. Furthermore, we methodically extracted 16 predictive variables associated with OC and computed the score for each sample utilizing the published coefficients. In each cohort, the predictive capability of all features for prognosis was evaluated using the C-index. To augment the clinical utility of the risk scores, we developed a nomogram utilizing the components derived from multivariate Cox (Multiv. Cox) regression. The C-index and calibration curves were plotted over time to assess accuracy, while the decision curve was employed to evaluate the clinical advantages for patients. The interpretation of the prediction model is performed by SHAP, which is a unified approach to calculate the contribution and influence of each feature toward the final predictions precisely.

### 2.6 Molecular characterization of immune-omics and comprehensive analysis of immunotherapy response based on molecular subtypes

Utilizing the Immunology-Oncology Bioinformatics Resources (IOBR) package, we gathered numerous earlier-published features pertaining to tumor microenvironment (TME) cell types, immunotherapy responses, immunosuppression, and immunological exclusion. Subsequently, we computed the enrichment scores for each sample employing a standardized methodology and conducted a thorough analysis of the immunological disparities between HRG and LRG patients. We analyzed the disparities in the distributions of tumor mutational burden (TMB), tumor neoantigen burden (TNB), and programmed death ligand 1 (PD-L1)–associated gene signatures (PPAGs, pertaining to tumor angiogenesis) between the two groups and subsequently reclassified the patients in conjunction with the risk scores. To evaluate the immunotherapy response, we integrated the Tumor Immune Phenotype (TIP) algorithm with the Tumor Immune Dysfunction and Exclusion (TIDE) algorithm.

### 2.7 Therapeutic screening

Following the stratification of patients into LRG and HRG, the GSEA algorithm was utilized in order to evaluate the activation status of oncogenic pathways. In order to acquire information regarding the drug sensitivity of Cancer Cell Lines (CCLs), the CTRP v.2.0 (https://portals.broadinstitute.org/ctrp) and the PRISM Reutilposing dataset (19Q4; https://depmap.org/portal/prism/) were employed. The dose-response curve was used to derive the area under the curve (AUC), which was then used as a measurement of drug sensitivity.

### 2.8 Patients

In order to create tissue microarrays, we examined retrospectively data from 180 patients diagnosed with OC and 20 patients who had oophorectomy procedures performed at Xijing Hospital, Affiliated with the Air Force Military Medical University for benign reasons between 2011 and 2017 (22, 23). Electronic medical records stored in the hospital’s database were used for retrieving the patients’ clinical data. Patients were considered for the study if they fulfilled the requirements that included the following: they had a pathologically diagnosed case of OC, normal ovarian samples, did not get any anticancer medication before the surgery, and did not experience any serious risk factors during the surgery that could impact their prognosis. For this group of patients, researchers used paraffin-embedded tissue samples. We additionally obtained new cancer and para-cancerous tissues from six additional OC patients. Patients diagnosed with OC had their malignant tumor stage assessed in accordance with the International Federation of Gynecology and Obstetrics’ recommendations (2023).

### 2.9 Cell culture and reagents

The Cell Bank of the Chinese Academy of Sciences in Shanghai, China, was the source of the OC cell lines OVCAR3 and SKOV3, which were taken from there. The cells were cultured in a medium that was composed of RPMI 1640 (PM150110, Procell, China) and was further enriched with 10% fetal bovine serum (FBS) (164210, Procell, Wuhan, China). Additionally, the medium contained penicillin G (100 U/ml, BYT-C0222, Beyotime, China) as well as streptomycin (100 μg/ml, BYT-C0222, Beyotime, China). At a temperature of 37°C, the culture was maintained in an incubator that was humidified and contained 5% CO_2_.

### 2.10 RNA processing and primer sequences

RNA extraction, cDNA synthesis, and RT-PCR reaction systems were carried out in accordance with the instructions of the RNA extraction, reverse transcription, and cycling reaction system kits provided by Polymerase. Primer synthesis was performed by Langjieke Company. The primer sequences used were: FXYD6 (forward: 5′‐ACCCTGAGGATTGGGGGAC‐3′, reverse: 5′‐CATTGGCGGTGATGAGGTT‐3′) and the internal reference TUBB/β-Tubulin (forward: 5′‐TGGACTCTGTTCGCTCAGGT‐3′, reverse: 5′‐TGCCTCCTTCCGTACCACAT‐3′).

### 2.11 Transfection

The design and synthesis of siRNA were carried out by ApexBio Company. After synthesis, transient transfection was performed using the transfection reagent Lipo3000. The transfection efficiency was confirmed by PCR and WB experiments to identify the optimal transfected small interfering RNA, which was used in subsequent experiments. The sequences of the interfering RNAs are as follows: SiFXYD6-1: 5′ -GCUGAAAAGGAGAAGGAAAT-3′; SiFXYD6-2: 5′ -AGGAAGCCCAGGUGGAGAAT-3′; SiFXYD6-3: 5′ -CCCAGAAAGCAGAGAACUGT-3′. The overexpression lentivirus was purchased from Shanghai GeneChem Co., Ltd., and the relevant transfection steps were performed according to the instructions.

### 2.12 Western blot analysis

The harvested tissues were homogenized and lysed with RIPA lysis solution (P0013B, Beyotime, China) for 20 minutes, which was followed by high-speed centrifugation at 13,500 rpm for 15 minutes. Cell lysates were additionally produced utilizing the RIPA technique. Following SDS-PAGE fractionation (PG112, Epizyme Biotech, Shanghai, China), protein samples were transferred to PVDF membranes (ISEQ00010, Millipore, USA). The FXYD6 antibody was acquired from Wuhan Sanying at a dilution of 1:1000; the β-tubulin antibody was obtained from Wuhan Sanying at a dilution of 1:2000; as well as the secondary antibody was sourced from Wuhan Sanying at a dilution of 1:10000.

### 2.13 Colony formation

A density of 500 cells per well was used to seed the OC cells onto 6-well plates. We changed the cell culture media every three days. After 12 days, the colonies were fixed for 15 minutes in 4% paraformaldehyde before being dyed with crystal violet. We used ImageJ to calculate the number of colonies in every well after photographing them.

### 2.14 Wound healing

We cultivated the OC cells in a medium supplemented with 10% FBS for 24 hours after seeding them in 6-well plates until 90% confluence was achieved. Afterwards, serum-free media was added to the wells after the cells were linearly scraped along their diameter employing a 1000 μL pipette tip. An inverted microscope was used to capture pictures of the wound at 0 and 48 hours after it had begun to heal, and the distance that had healed was then determined.

### 2.15 Cell viability

We seeded 96-well plates with the cancer cell solution, distributing 6,000 cells per well. In accordance with the guidelines provided by GlpBio, USA’s Dojindo Cell Counting Kit-8 (CCK-8, GK10001, after attachment) was used to assess cell viability at 0, 24, 48, and 72 hours. Cell confluence throughout the test was kept between 80 and 90% by carefully seeding the appropriate amount of OVCAR3 and SKOV3 cells into 96-well cell culture plates. A cytotoxicity detection kit for lactate dehydrogenase (LDH) was used to test the levels (LDH Cytotoxicity Detection Kit, C0016, Beyotime, China).

### 2.16 Animal model

Female BALB/c nude mice were sourced from Beijing Vital River Laboratory Animal Technology Co., Ltd. for the study when they were 4 to 5 weeks old. The Air Force Medical University’s Institutional Animal Care and Use Committee ensured that all experiments involving animals followed its guidelines. Mice received subcutaneous injections of 1×10^6^ OC cells in 100 μL of a solution (PBS: Matrigel = 1:1) in the axillary area. All mice were slaughtered 30 days post-inoculation, and the tumor-bearing tissues from each mouse were harvested. A portion of the tumor tissues was preserved in 4% paraformaldehyde and subsequently embedded in paraffin for immunohistochemistry (IHC) staining.

### 2.17 Immunohistochemistry staining

IHC staining was performed subsequent to the deparaffinization of tissue slides in xylene and rehydration in alcohol, subsequently followed by treatment with 3% H2O2 to inactivate endogenous peroxidase. An antigen repair treatment utilizing a microwave was subsequently conducted in a 0.1M sodium citrate buffer at pH 6.0. Following a 30-minute incubation at room temperature with 5% normal goat serum for blocking, primary antibodies were subsequently incubated overnight at 4°C. The nuclear visualization was achieved by first incubating the poly-HRP secondary antibody for 60 min at room temperature without light and then counterstaining with hematoxylin. Our hospital’s pathologists reviewed the stained images captured by the Caseviewer scanner. We classified FXYD6 expression as either 0 “negative,” 1 “weak,” 2 “moderate,” or 3 “strong” based on the staining intensity. One ranged from 0% to 25%, and four ranged from 76% to 100% in the positive proportion area. The FXYD6 expression findings were defined as scores between 0 and 12. All necessary ethical approvals have been obtained, and the study project is being carried out in full accordance with established ethical guidelines (24).

### 2.18 Ethics

The Ethics Committee at Xijing Hospital, affiliated with Air Force Medical University, authorized the utilization of retrospective information concerning patients. Informed permission in writing was acquired from all subjects.

### 2.19 Statistical analysis

The unpaired Student’s t-test was utilized for group comparisons in the event that the variables were regularly distributed. On the other hand, the Wilcoxon rank-sum test was utilized in the event that the variables were not normally distributed. Whenever comparing more than two groups, the Kruskal-Wallis test was utilized for non-parametric variables, while the one-way analysis of variance was utilized for parametric variables. For the purpose of analyzing contingency tables, a two-sided Fisher’s exact test was performed. The survminer package’s “surv-cutpoint” function established the threshold for molecular categorization. It was possible to classify patients in each cohort into HRG and LRG based on the optimally chosen log-rank statistic, which mitigated the computational batch impact. We performed differential expression analysis using the limma tool and multi-omics clustering using the MOVICS package. We used R v.4.1.0 for all of our statistical analysis. The images of the *in vitro* studies were created using GraphPad Prism 9. Each experiment was carried out three times, and the means ± standard deviations (SD) of the continuous variables were used to represent themselves. Statistical significance was defined as a P-value below 0.05.

## 3 Results

### 3.1 Multi-omics consensus prognostic related molecular subtypes of OC

**Figure 1:**
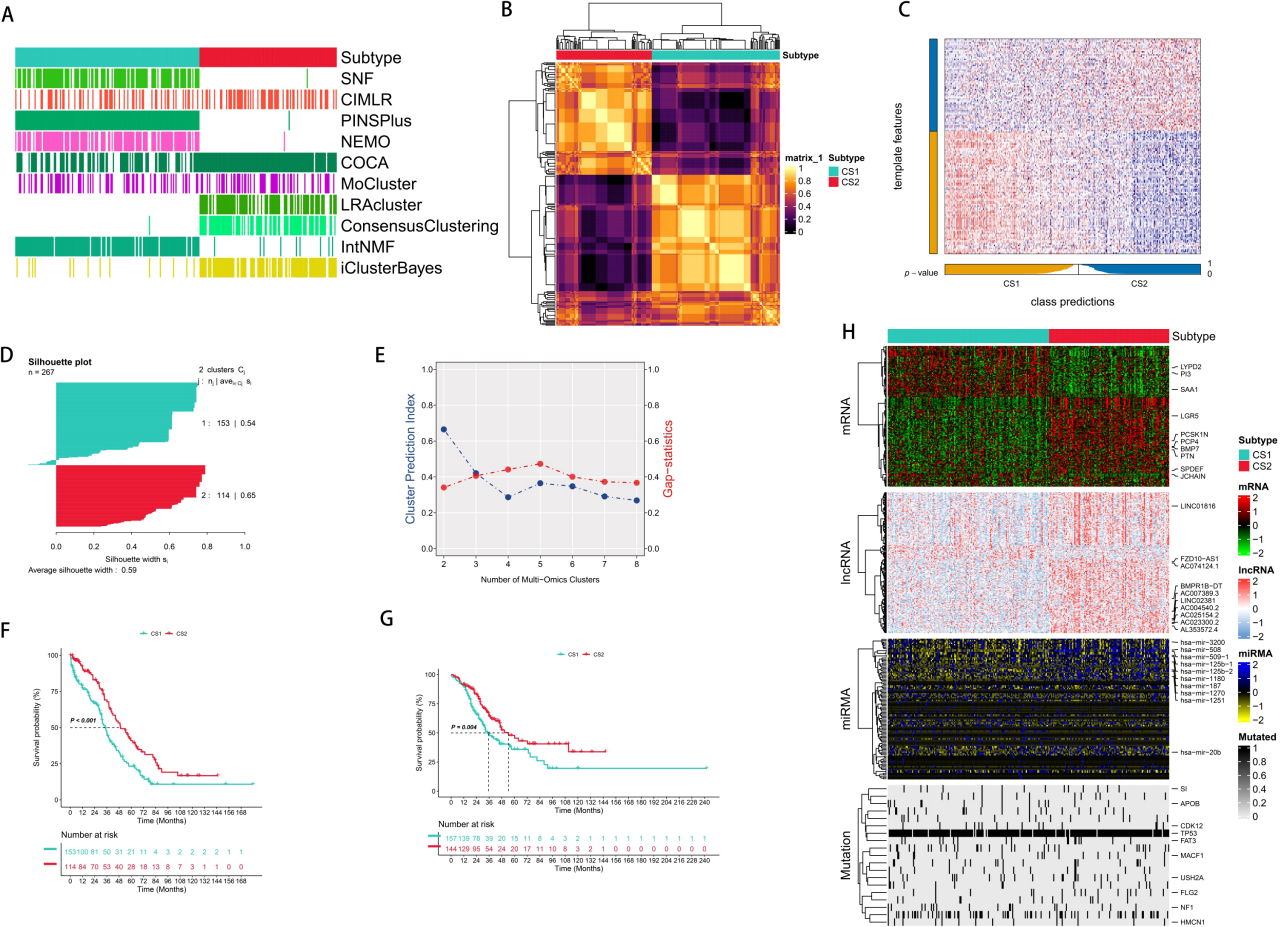
Integrated common subtypes of ovarian cancer multi-omics. **(A)** 10 multi-omics clustering methods were used to cluster ovarian cancer patients. **(B)** Consensus clustering matrix of two new prognostic subtypes based on 10 algorithms in the TCGA training cohort. **(C)** Validation of the new prognostic subtypes in the GEO integrated cohort. **(D)** Silhouette score was calculated to measure the similarity among samples. **(E)** Cluster prediction index and gap statistical analysis of the new prognostic subgroups. **(F)** The survival outcomes of the two subtypes were different in the TCGA training cohort. **(G)** Validation of the survival outcomes of the two subtypes in the GEO integrated cohort. **(H)** Comprehensive heatmap of the new prognostic subtypes (mRNA, lncRNA, miRNA, mutated genes).

After effectively preprocessing all the data, we further validated our results by employing PCA to analyze the data before and after handling the batch effect (**Supplementary Figure S1**). We independently identified two subtypes from ten multi-omics integrated clustering algorithms (iClusterBayes, moCluster, Cancer Integration via MultIkernel LeaRning (CIMLR), Integrative Clustering of Multiple Genomic Dataset (IntNMF), ConsensusClustering, Cluster-Of-Cluster-Assignments (COCA), Neighborhood-based Multi-omics Clustering (NEMO), PINSPlus, Similarity Network Fusion (SNF), and Landscape Reconstruction Algorithm (LRA)) (**Figures 1A–C**), and comprehensively referred to clustering prediction metrics and gap statistic analysis to determine the number of molecular classifications (**Figures 1D, E**). Our classification system is closely associated with OS (TCGA: P < 0.01; validation set: P = 0.004). Notably, Molecular Subtype 2 (CS2) exhibits the best survival outcome (**Figures 1F, G**).

We used the consensus integration approach to integrate the clustering findings with the specific molecular expression patterns of the transcriptome (mRNA, lncRNA, as well as miRNA) and somatic mutations after we obtained the stable prognostic molecular classification (**Figure 1H**). We used omics data from multiple dimensions to identify the 1,500 genes with the greatest levels of variation. Subsequently, as a first step in the clustering process, genes associated with prognosis were eliminated using Cox regression analysis. Mutation frequency was used to filter candidate genes for mutation data. Excluding the core feature genes with the greatest C-index, we used all combinations of ML algorithms (Enet, Stepwise Cox, CoxBoost, Ridge Regression, RSF, GBM, Survival-SVM, LASSO, SuperPC, plsRcox, and Stepwise Cox) to further discover stable prognostic genes based on subtypes. The molecular categorization of OC was subsequently constructed utilizing RSF. Thoroughly established were the connections between molecular categorization and prospective therapeutic medications, tumor immune microenvironment, immunotherapy response, as well as prognosis.

### 3.2 Characterization of integrated consensus molecular subtypes in OC

Molecular subtypes based on genes related to endometrial cancer have been applied in clinical practice and achieved good results. The incidence and mortality of OC are increasing year by year, and most cases of OC are already at an advanced stage when diagnosed. Moreover, previous research on the molecular classification of OC mainly focused on single omics or clinical test indicators. Therefore, continuous exploration of new molecular subtypes is still needed. Based on the previous analysis, we generated two prognostic molecular subtypes and explored the characteristics of these molecular subtypes.

**Figure 2:**
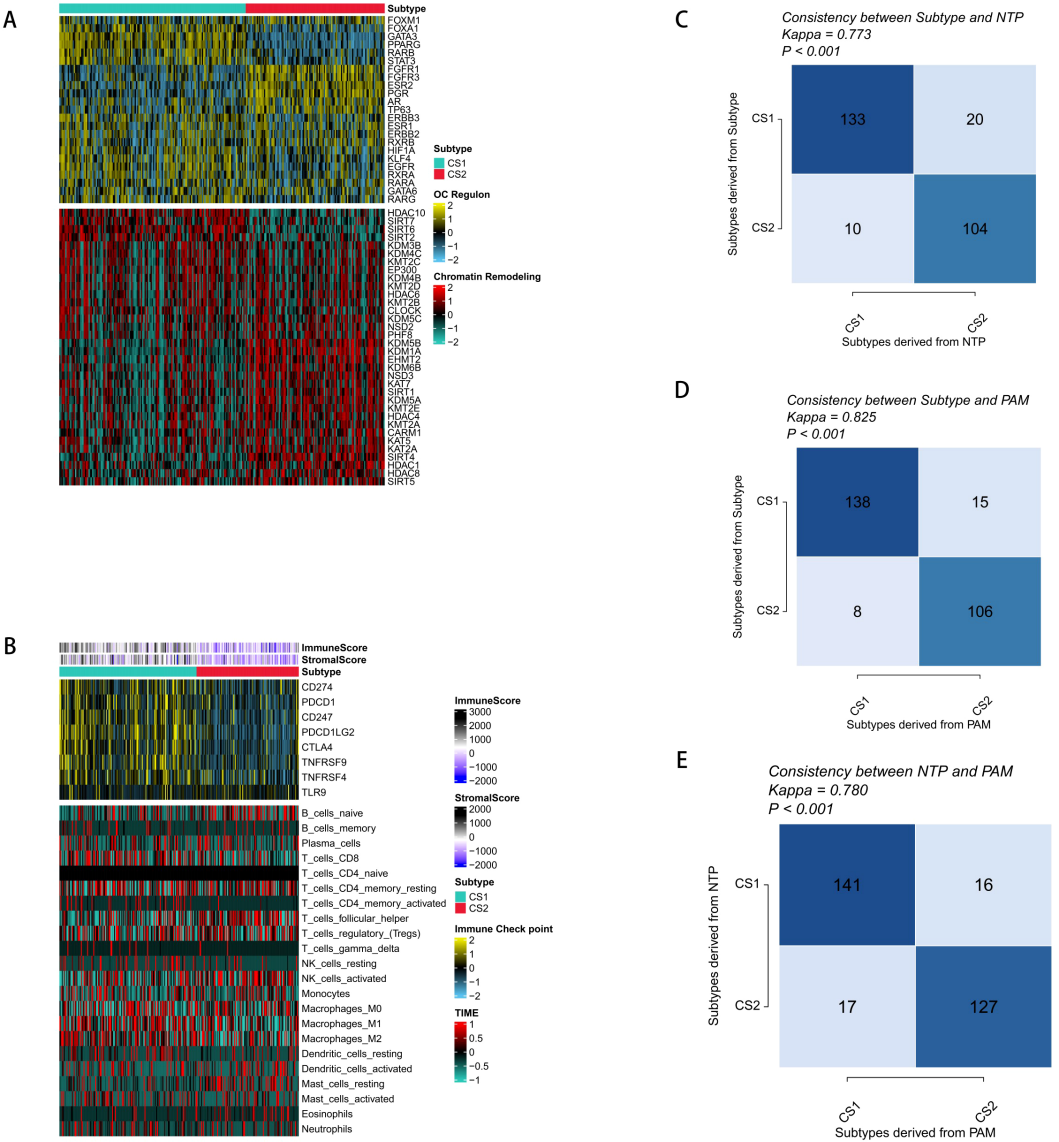
Molecular profiles and validation of the new molecular subtypes of ovarian cancer. **(A)** Regulatory activity profiles of 23 transcription factors (top) and potential regulators related to chromatin remodeling for the two subtypes (bottom). **(B)** Immune signature profiles in the TCGA training set. The immune enrichment scores and microenvironment scores of tumor-infiltrating lymphocytes are shown at the top of the heatmap. The upper figure shows the expression differences of classic immune checkpoint genes, and the lower figure shows the enrichment levels of 24 immune cells related to the tumor microenvironment. **(C)** Consistency between the new subtypes and NTP in the TCGA cohort. **(D)** Consistency between the new subtypes and PAM in the TCGA cohort. **(E)** Consistency between NTP and PAM in the GEO validation cohort.

To further study the differences in the transcriptome, we analyzed the potential regulators related to cancerous chromatin remodeling and 23 transcription factors (TFs) associated with cancer. The close correlation between regulator activity and molecular classification confirmed the biological relevance of the molecular classification. In the CS1 subtype, FOXA1, GATA3, PPARG, RARB, and EGFR were significantly activated, while FGFG1, FGFR3, PAG, ESR2, etc. were significantly activated in the CS2 subtype (**Figure 2A**). The role of epigenetics in tumors has received increasing attention, and the activity profiles of regulators related to tumor chromatin remodeling further highlighted the potential patterns of differential regulation among CS subtypes.

Our analysis of the CS1 subtype’s levels of microenvironmental cell infiltration revealed that both the immunological score as well as the microenvironment score were significantly greater than those of the CS2 subtype. Additionally, genes related to immunotherapy were significantly enriched in the CS1 subtype, which is important because tumor immunity plays a crucial part in tumorigenesis as well as progression. It was thus speculated that immunotherapy might have a better effect on the CS1 subgroup (**Figure 2B**). However, the distribution of immune cell infiltration varies greatly among molecular subtypes. Combining our previous research and actual clinical observation results, OC belongs to the immune-desert type of tumor, and the effect of immunotherapy is not obvious. Therefore, it is still necessary to explore better therapeutic targets.

To determine the stability of the molecular subtypes, we performed the same classification in the validation cohort and found that patients could be clearly divided into two subgroups, and the survival outcome of CS2 was still better than that of CS1 (**Figure 1C**). To further determine the effectiveness of the subtypes, we used NTP to classify each sample in the external cohort into one of the identified CS subtypes and also evaluated the consistency between CS and the NTP and K-medoids (PAM) algorithms (p < 0.005) (**Figures 2C–E**).

### 3.3 Construction of molecular subtypes

**Figure 3:**
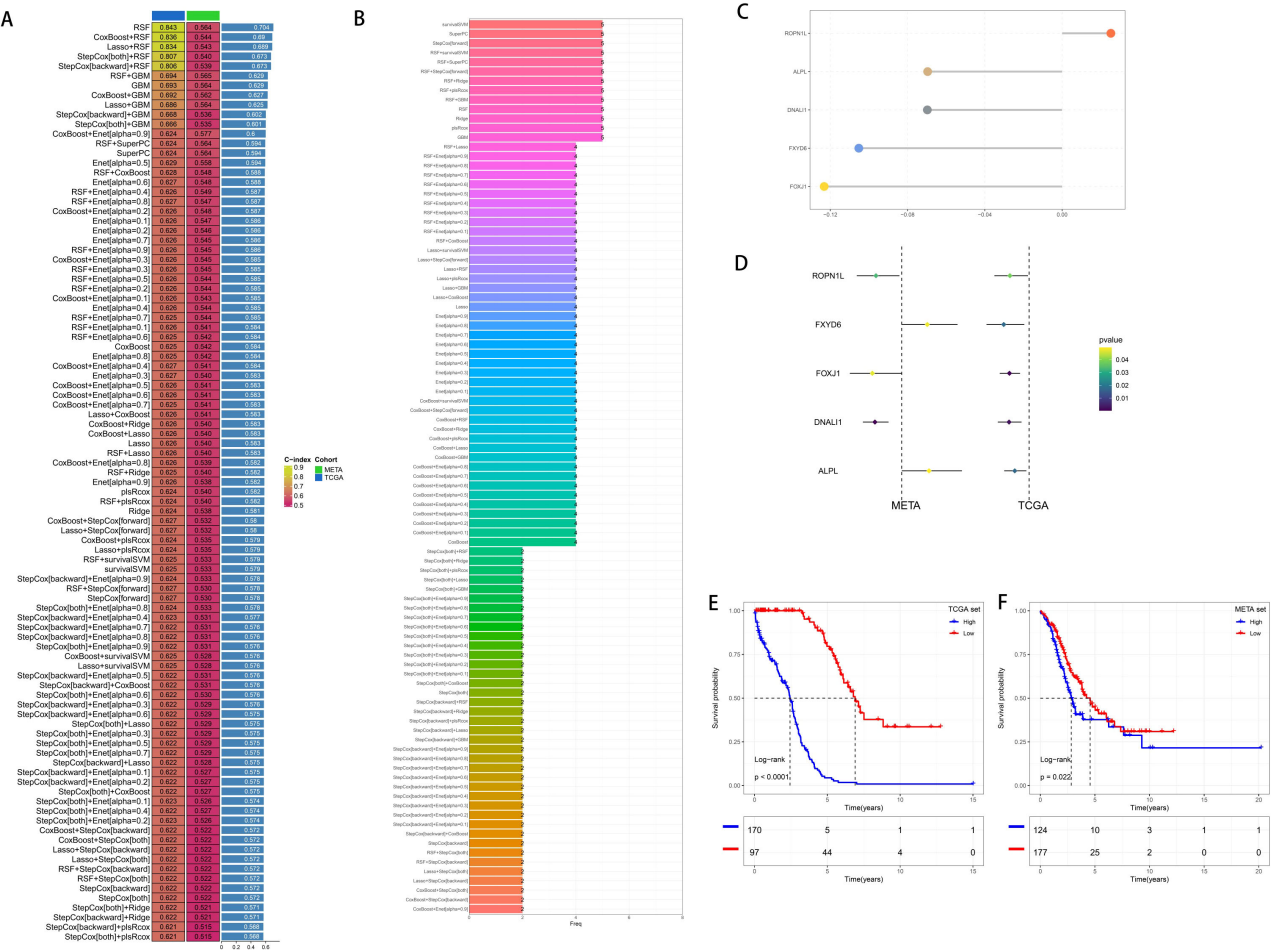
Generation and Prognostic Value of Risk Scores. **(A)** By integrating and constructing a comprehensive computational framework, combinations of 95 machine learning algorithms were generated. The C-index of each model was calculated from the TCGA cohort and the GEO integrated cohort (META cohort) and sorted according to the average C-index. **(B)** The number of genes constituting the combinations of 95 machine learning algorithms. **(C)** Hub genes selected by the RSF algorithm. **(D)** Univariate Cox regression results in the training cohort and the validation cohort. **(E)** Survival analysis of patients with high and low risk scores in the training cohort. **(F)** Survival analysis of patients with high and low risk scores in the validation cohort.

We evaluated five stable DEGs from the TCGA and GEO for a significant association between expression and OS using univariate (Univ. Cox) and multivariate (Multiv. Cox) regression models. Subsequently, these stable differentially expressed survival genes were incorporated into an integrated framework to perform the construction of molecular subtypes. To evaluate the predictive accuracy of all the models, we created standard models in the TCGA training cohort using 95 different algorithm combinations and averaged their C-indexes over all the cohorts (**Figures 3A, B**). The 95 models demonstrated that RSF algorithm achieved the highest average C-index in the development of the final model, which comprised five core genes (**Figures 3C, D**). Then, we calculated the risk scores of each sample in all cohorts. Patients with high risks had poorer clinical outcomes (**Figures 3E, F**).

### 3.4 Comparison of prognosis and metastasis characteristics of molecular subtypes

**Figure 4:**
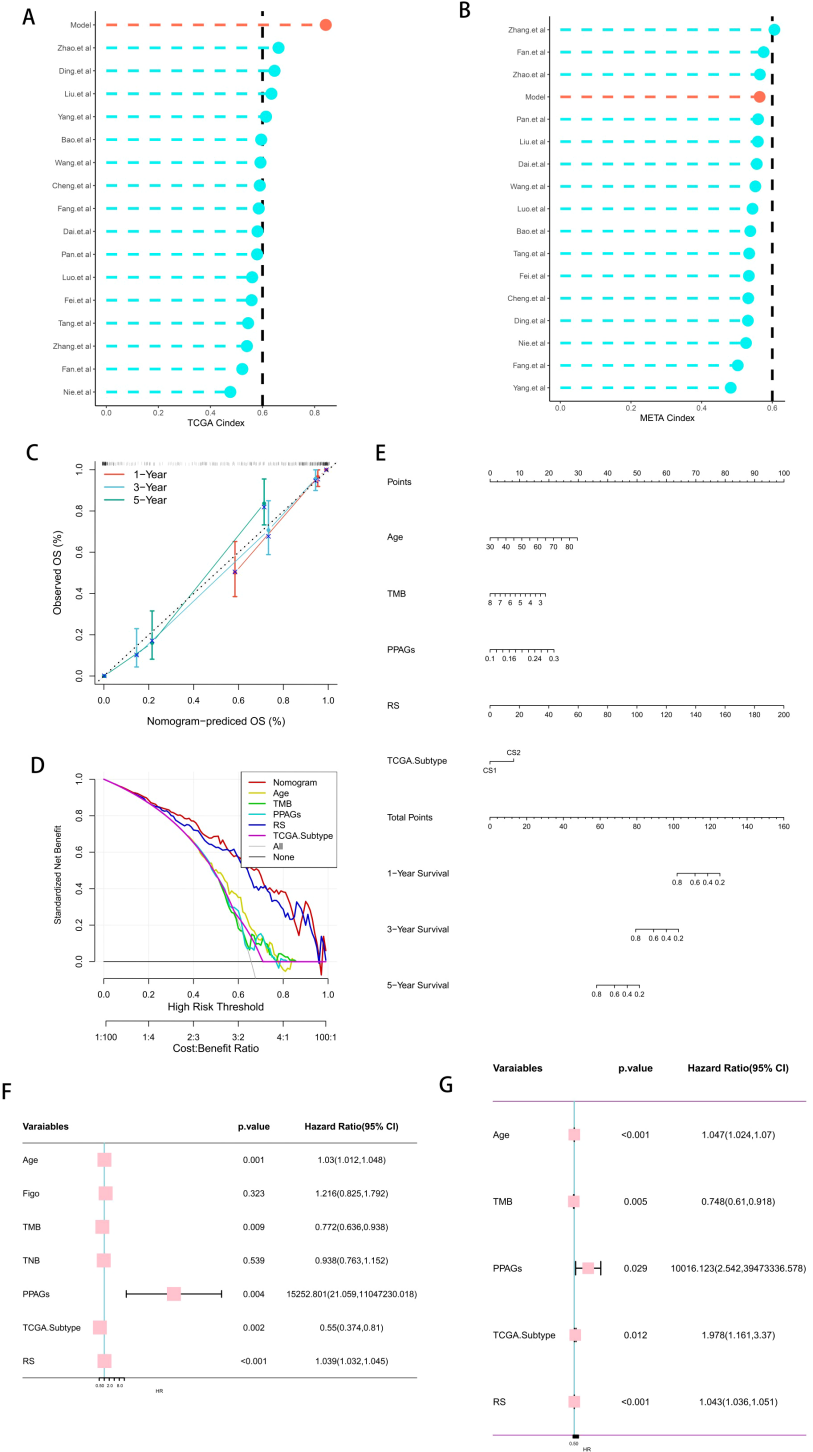
Clinical Utility Value of Risk Scores. **(A, B)** Comparison of the risk scores with other published models in the training cohort and the validation cohort. **(C)** Calibration curve of the comprehensive nomogram. **(D)** Decision curve of the comprehensive nomogram. **(E)** Comprehensive nomogram based on the risk scores. **(F, G)** Univariate and multivariate analyses of the risk scores and other clinical indicators to determine the value of the risk scores.

Current developments in next-generation sequencing technologies have led to the widespread reporting of various gene expression-based prognostic markers. To thoroughly compare molecular subtypes with other signatures, we rigorously reviewed pertinent literature published in the last five years and eventually included 16 distinct signatures in our study (**Figures 4A, B**). These markers are linked to several biological processes, including immunotherapy response, immunological infiltration, and ferroptosis. The molecular subtypes had a C-index performance that surpassed nearly all models identified in the present investigation (**Figures 4A, B**).

In light of the clinical application possibilities associated with molecular categorization, we identified promising independent prognostic markers for OC by independent prognostic analysis and synthesized them to develop a full nomogram presented as a web calculator. **Figures 4C-E** show that Decision Curve Analysis (DCA) indicated that the nomogram’s therapeutic benefits for patients were significantly higher than those of using CMLS alone, and the calibration curve demonstrated that the nomogram’s accuracy was in line with reality. After that, we verified the risk score’s independent prognostic significance using Univ. Cox and Multiv. Cox analyses. The risk score was found to be a potential independent prognostic factor in both Univ. Cox and Multiv. Cox analyses (**Figures 4F, G**).

### 3.5 Molecular subtype related immune characteristics

Utilizing the Immunology-Oncology Bioinformatics Resources (IOBR) R package, we performed an extensive investigation of the TME of OC and noted that the infiltration levels of immune cells (including T cells, B cells, and macrophages) in HRG were elevated compared to those in LRG (**Supplementary Figure S2A**). However, an analysis of the immunosuppressive state revealed that myeloid-derived suppressor cells played a more significant role in immunotherapy in the HRG. Therefore, we further analyzed the pathways related to immune escape and found that the immune escape state was significantly increased in HRG compared to that in LRG. Genes related to mismatch repair were mainly enriched in LRG (**Supplementary Figures S2B–D**). The results aligned with our prior study, indicating that HRG is associated with a worse prognosis, possibly linked to immunosuppression and immune evasion, suggesting that HRG OC patients are more prone to being categorized as “cold tumors.” Subsequently, we further examined the differences of immune checkpoint, T cell depletion and immune escape cytokine related marker genes in the high-low risk group, and the results indicated that the expression of immune checkpoint marker was higher in the low-score risk group, while the expression of immune cell depletion and immune escape cytokine was higher in the high-risk group(**Supplementary Figure S2E**). These results are consistent with previous findings that patients in the high-risk group may benefit less from immunotherapy.

TMB and TNB are acknowledged biomarkers for assessing patient outcomes after immunotherapy. In addition, we also analyzed the role of tumor angiogenesis-related pathways in immunotherapy. The results indicated that the LRG had a higher TMB score; however, no significant difference was seen between the groups about the TNB. These results could be part of the reasons for the better prognosis of the LRG (**Supplementary Figure S2F**). Angiogenesis in tumors was more pronounced in the HRG, and there was a strong correlation between the high immune cell infiltration and the risk of programmed death ligand 1 (PD-L1)-associated gene signatures (PPAGs), which elevated in parallel with the risk score (**Supplementary Figures S2G, H**). In addition to TMB, TNB, and PPAGs, survival analysis suggested that the risk score could be a useful supplementary factor for patient prediction. Patients with OC had a better chance of survival when their risk score was lower, their TMB or TNB were higher, or their PPAGs were lower (**Supplementary Figures S2I–K**).

### 3.6 Molecular subsets for immunotherapy and potential therapeutic drug screening

To deeply explore the differential patterns of molecular subtypes, we systematically investigated the relationships between the genes used for subgroup construction and copy number variation; gene set variation score, methylation, and clinical stage in common malignant tumors of the female gynecological system. The research findings demonstrated that both the copy number variation and the variation rate of variant gene expression were the highest in OC (**Supplementary Figures S3A–C**). The genes most closely related to the clinical stage were ALPL and FXYD6 (**Supplementary Figure S3D**). The degree of methylation variation was most significant in endometrial cancer (**Supplementary Figure S3E**). The gene set variation score showed a negative correlation with the clinical stage in OC (**Supplementary Figure S3F**). Analysis of the activation and inhibition of targets revealed that FXYD6 exhibited the richest target characteristics (**Supplementary Figure S3G**). DNA damage and hormone receptors were significantly correlated in gynecological tumors (**Supplementary Figure S3H**). Analysis of immune differences with the core genes as a reference showed that the distribution of immune cells in OC had the most significant difference among these classifications (**Supplementary Figure S3I**).

Furthermore, we determined the Tracking Tumor Immunophenotype (TIP) to investigate possible biological attributes linked to molecular subtypes. We found that the differences between the HRG and LRG were mainly concentrated in step 4 (recruitment of tumor immune infiltrating cells), which is consistent with our previous research (**Supplementary Figure S4A**).

Furthermore, we employed the TIDE methodology to assess patients’ outcomes after immunotherapy. We found that the difference between the two groups was not significant, but the LRG generally had a higher response to immunosuppressive agents than the HRG (**Supplementary Figure S4B**). Combined with the previous analysis results, it is overall found that the prognosis of the HRG is poor. To improve the prognosis level of the HRG, we performed GSEA pathway enrichment analysis on the HRG. The findings indicated that the difference pathways were mostly focused on epithelial-mesenchymal transition, NF-κB signaling, immunological inflammation, hypoxia, and other pathways (**Supplementary Figure S4C**). Subsequently, we employed the Cancer Therapeutics Response Portal (CTRP) and the Parallel Resistance in Mixtures Screen (PRISM) to evaluate possible therapy agents for patients in the HRG. To validate the robustness of our strategy, we employed cisplatin, a commonly utilized medication for bladder cancer, to ascertain whether the sensitivity obtained from the algorithm aligns with established clinical practice. Studies have established that ERCC1 serves as a predictive biomarker for individuals undergoing platinum-based chemotherapy. From this, we derived the following conclusions: in line with earlier research, patients who had decreased ERCC1 levels demonstrated stronger responsiveness to cisplatin, suggesting that this drug may improve chemotherapy outcomes for such individuals (**Supplementary Figure S4D**). We then proceeded to thoroughly investigate possible medications for HRG patients. Finally, we found that the HRG had a higher sensitivity to tyrosine kinase inhibitors, which can be used as one of the candidate drugs to improve the prognosis of patients in the HRG (**Supplementary Figures S4E, F**).

### 3.7 Functional analysis of FXYD6 *in vitro*

**Figure 5:**
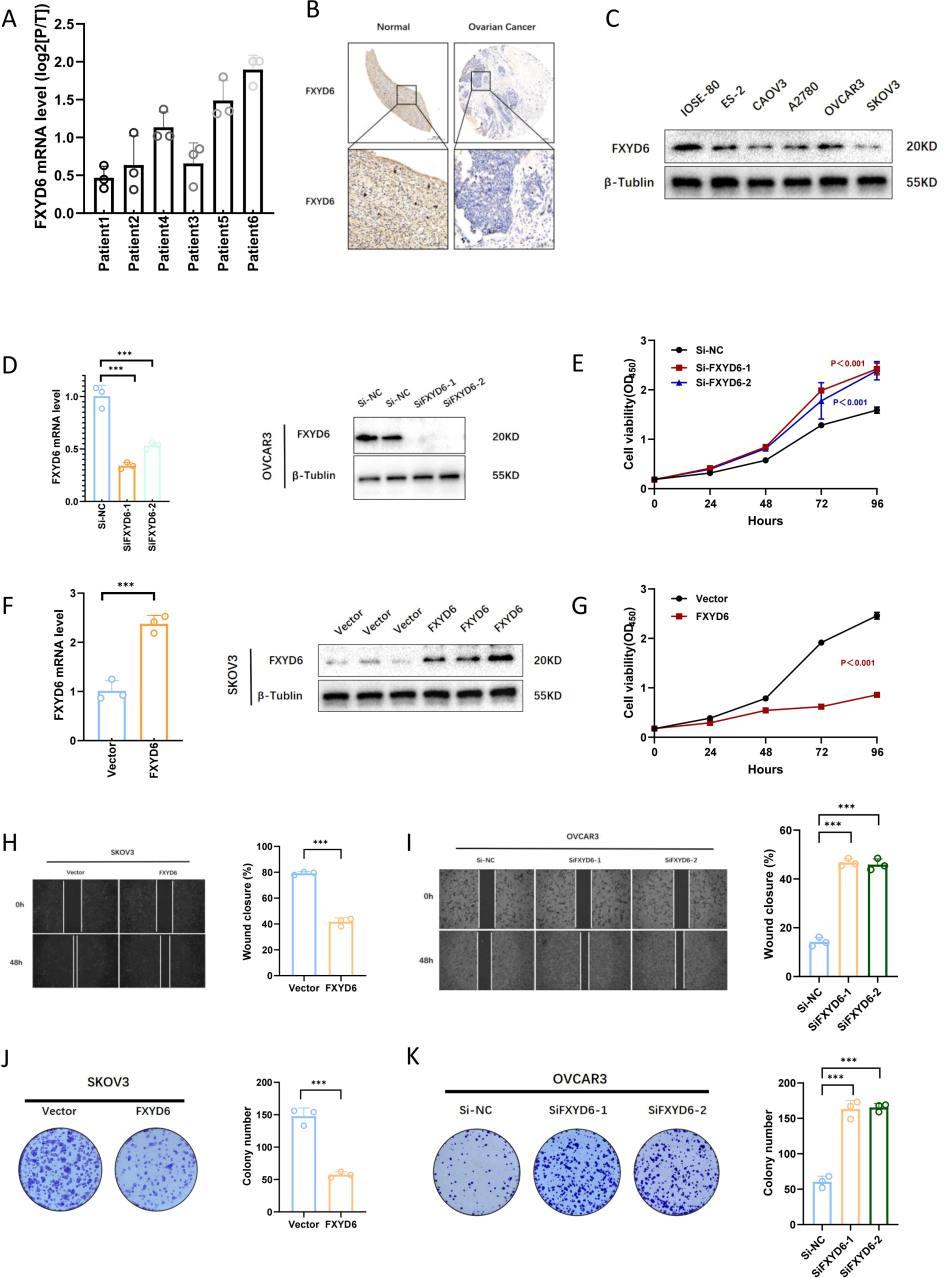
Validation of FXYD6 Expression and *In Vitro* Functional Analysis. **(A)** Changes in FXYD6 expression in paracancerous tissues and tumor tissues of 6 ovarian cancer patients at the RNA level. **(B)** Differences in FXYD6 expression between tumor tissues and normal ovarian tissues at the protein level. **(C)** Differences in FXYD6 expression between normal ovarian epithelial cell lines and ovarian cancer cell lines. **(D)** Detection of the effect of knocking down FXYD6 by small interfering RNA. **(E)** Detection of changes in the proliferation ability of ovarian cancer cells after knocking down FXYD6 by CCK8 assay. **(F)** Detection of the effect of overexpressing FXYD6 by lentivirus. **(G)** Detection of changes in the proliferation ability of ovarian cancer cells after overexpressing FXYD6 by CCK8 assay. **(H, I)** Detection of the proliferation and migration abilities of cancer cells after knocking down and overexpressing FXYD6 by wound healing assay. **(J, K)** Detection of the *in vitro* proliferation abilities of cancer cells after knocking down and overexpressing FXYD6 by colony formation assay. ***: Indicates that P < 0.001.

Tumor metabolic reprogramming has received increasing attention. We retrieved from the core genes and combined them with the correlation of previous clinical data and found that FXYD6 plays an important role in tumor metabolism and the electron transport respiratory chain. In addition, we evaluated the predictive power of individual genes in the predictive model through SHAP (SHapley Additive exPlanations) and found that FXYD6 contributed the most (**Supplementary Figure S4G**). Therefore, we systematically studied the gene function of FXYD6. The literature search revealed that the function of FXYD6 in OC is currently poorly understood. We explored its role in tumor cell proliferation and migration. In the first step, we used RT-PCR to identify alterations in FXYD6 expression in carcinoma tissues. Paracancerous tissues showed a significantly greater level compared to cancerous tissue at the transcriptional level (**Figure 5A**). Subsequently, we used tissue microarrays of 200 cases to explore the differences in expression changes at the protein level. Compared with normal ovarian tissues, the expression at the protein level in tumor tissues was significantly lower than that in normal ovarian tissues. The above results suggest that the content of FXYD6 is higher in normal tissues and lower in tumor tissues (**Figure 5B**).

To explore the functional role of FXYD6 in OC, we used siRNA for gene knockdown and lentivirus for gene overexpression. Firstly, we detected the expression differences of FXYD6 in normal ovarian epithelium and OC cell lines by Western blot. The results suggested that its expression was lower in OC. Based on the expression levels, we selected OVCAR3 with the highest expression as the knockdown cell line and SKOV3 with the lowest expression as the overexpression cell line (**Figure 5C**). We utilized CCK8, plate cloning, and scratch tests to examine the effects of gene interference on cell proliferation and migration following PCR, and Western blotting confirmed the impacts of gene interference (**Figures 5D, F**). The findings showed that OC cell proliferation and migratory capacities were enhanced following gene knockdown and diminished following gene overexpression.

Specifically, in the CCK8 cell proliferation assay, it was found that the proliferation ability of OC cells was enhanced after SiFXYD6 treatment (**Figure 5E**); after overexpression, the proliferation ability of OC cells was weakened, and the cell growth was very slow (**Figure 5G**). The wound healing assay also found that the wound healing ability of OC cells was enhanced after SiFXYD6 treatment (**Figure 5I**); after overexpression, the wound healing ability of OC cells was weakened (**Figure 5H**). **Figure 5K** shows that the control group had significantly weaker colony formation capacity following SiFXYD6 treatment, while **Figure 5J** shows that overexpression significantly impaired the colony formation capacity of OC cells. The findings of cancer cell cloning formation capacity testing demonstrated that both the number and size of colonies formed by OC cells were significantly greater following SiFXYD6 treatment.

**Figure 6:**
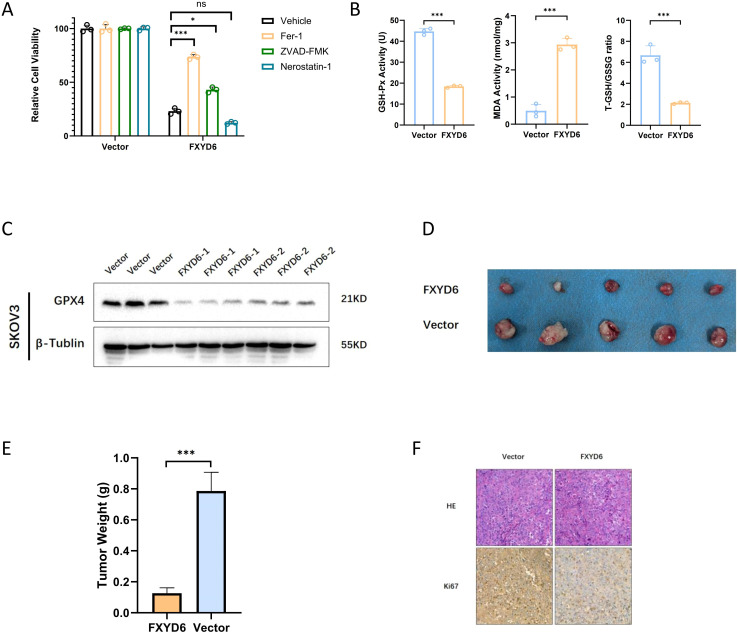
FXYD6 Protects Cancer Cells from Ferroptosis and *In Vivo* Functional Analysis. **(A)** The viability of cells with overexpressed FXYD6 was detected after culturing for 24 hours with or without 1 μM Fer-1, 2 μM Necrostatin-1, and 5 μM ZVAD-FMK. **(B)** Detection of ferroptosis-related indicators including GSH-Px, MDA, and T-GSH/GSSG. **(C)** Detection of the ferroptosis marker GPX4 in overexpressed cells. **(D)** Tumorigenesis experiment in nude mice using overexpressed cells. **(E)** Differences in the mass of tumor-bearing tissues. **(F)** Detection of the proliferation indicator Ki-67 in tumor-bearing tissues. *: Indicates that P < 0.05; ***: Indicates that P < 0.001. ns, not significant.

In addition, during the process of cell culture, we also observed that a large number of cells died after FXYD6 overexpression, which might be one of the reasons for these phenomena. Therefore, we treated the overexpressed OC cells with 1 μM ferroptosis inhibitor, 5 μM apoptosis inhibitor, and 2 μM necroptosis inhibitor to explore the possible causes of cell death. The research findings demonstrated that the ferroptosis inhibitor could significantly alleviate the cell death caused by gene overexpression, and the apoptosis inhibitor could also alleviate it, but to a lesser extent than the ferroptosis inhibitor (**Figure 6A**). Therefore, we speculated that the cell death caused by gene overexpression was largely due to ferroptosis and partly due to apoptosis.

Based on the above results, we then detected the changes in ferroptosis-related indicators after overexpression. The research findings demonstrated that the level of lipid peroxidation in cells increased after overexpression; the T-GSH/GSSG level was significantly reduced, that is, the content of reduced glutathione decreased, and the content of oxidized glutathione increased; and the content of GSH-Px decreased (**Figure 6B**). Subsequently, we detected the level of the ferroptosis marker GPX4 at the protein level, and the results also showed that the level of the ferroptosis marker was significantly reduced after gene overexpression, suggesting that ferroptosis occurred in OC cells after overexpression (**Figure 6C**).

Subsequently, we investigated the cell proliferation and growth abilities *in vitro* and conducted an *in vivo* tumor-bearing experiment in nude mice. Each nude mouse was inoculated with 1×10^6^ cells in the axillary region, and the tumor tissues were collected for comparison 3 weeks after tumor formation. The research findings demonstrated that the tumorigenic ability of the cells decreased after overexpression; the weight of the tumor-bearing tissues was also significantly smaller than that of the control group (**Figures 6D, E**). Detection of the cell proliferation ability revealed that the Ki67 level in the overexpressed tumor tissues was lower than that in the control group (**Figure 6F**).

## 4 Discussion

Methylation, mutation, as well as histone modification are among the many genetic and epigenetic processes that precisely regulate gene expression (25). A thorough investigation of patients’ multi-omics data will enhance our comprehension of disease-specific regulatory systems (25, 26). Nevertheless, until now, the majority of research studies conducted have mostly concentrated on individual omics research (27). The selection of omics clustering techniques is predominantly influenced by individual preference, and as their application broadens, the constraints of specific methods become more apparent. Our research intends to address this deficiency. We used the most recent 10 clustering techniques and discovered two prognostic subgroups of OC with distinct characteristics, which may hold significant significance for the precise stratified therapy of patients. The novel subtypes have demonstrated stability across various cohorts. Our classification has the potential to enhance conventional classification strategies.

ML algorithms are now efficient techniques for analyzing multi-omics data (28). To elucidate the distinctions in molecular characteristics among various prognostic subtypes and enhance clinical applicability, we utilized the GEO as the validation cohort and TCGA as the training cohort, subsequently identifying the optimal predictive risk score via 95 algorithmic combinations to mitigate the constraints imposed by algorithm selection. Presently, the integration of technologies like artificial intelligence with extensive biological big data presents overfitting as a significant concern in the model development process. Models that excel in the training set often struggle to generalize adequately to different validation sets. To address the challenges associated with overfitting the training set, we utilized the average C-index of multiple validation cohorts as the ranking criterion. The study revealed that the RSF algorithm had robust prediction capability in both the training and validation sets. Similarly, we observed that, compared with other published signatures, the carefully screened prognostic risk score showed a powerful prognostic value in each cohort.

Utilizing the Gene Set Enrichment Analysis (GSEA) algorithm and the Immunology-Oncology Bioinformatics Utilizing the IOBR R package, we analyzed the enrichment of various immune-related variables between the two groups. We discovered that various carcinogenic pathways in the high prognostic score cohort were significantly active, and the cold tumor phenotype was more prevalent.

The survival analysis of the low prognostic score also showed a better prognosis result. Two prevalent predictive instruments, TIDE and Subclass Mapping, demonstrated improved immunotherapy response in the LRG, aligning with our analysis and suggesting that the risk score may facilitate the prompt detection of individuals responsive to immunotherapy. Furthermore, we comprehensively evaluated possible therapeutic agents utilizing an extensive screening strategy demonstrated to be beneficial in prior research. Ultimately, tyrosine kinase inhibitors were evaluated as potential therapeutic agents for the LRG.

FXYD6, a member of the FXYD family, is defined by a highly conserved FXYD motif (Phe-X-Tyr-Asp, where X represents any amino acid) and serves as the sixth identified member of this ion transport regulator family (29). The mammalian FXYD family has seven members, FXYD1-7, that compose type I transmembrane proteins exhibiting similarities in both protein structure and function. FXYD regulates the Na+/K+-ATPase, situated on the cell membrane and consisting of α and β subunits (30). FXYDs modulate the comprehensive enzymatic kinetic characteristics of Na+/K+-ATPase by modifying the transport rate and affinity for Na+ and K+ ions (31, 32). Recent findings suggest that FXYD proteins stabilize the active conformation of Na+/K+-ATPase through direct interaction (30). Several investigations have indicated that FXYD6 is significantly expressed in cholangiocarcinoma and hepatocellular carcinoma (29, 33). Recent investigations have shown that FXYD1, FXYD5, and FXYD7 can enhance migration as well as invasion of OC cells (34). Nonetheless, the role and mechanism of FXYD6 in ovarian cancer have not been documented until now.

Studies have reported that FXYD6 can participate in the electron transfer and ion binding of the Na+/K+-ATPase, thereby affecting the activity of the enzyme. Regarding its function, we speculated that interfering with the expression of FXYD6 could influence the malignant behaviors of OC cells. We first evaluated the changes in its expression in OC. The research findings demonstrated that both the RNA and protein levels of FXYD6 were lowly expressed in OC. Consistent with previous studies, excessive expression of FXYD6 could inhibit the growth of tumor cells, and this phenomenon might be related to its function. As a regulator of the Na+/K+-ATPase, FXYD6 has the function of inhibiting sodium ion transport (32). Excessive expression would cause an imbalance of intracellular sodium ions, thus triggering a series of reactions. Our results also reached the same conclusion. After knocking down its expression, the growth and proliferation of OC cells were accelerated. After overexpression, its growth was significantly inhibited, and a large number of cells died.

To explore the causes of cell death after gene overexpression, we used apoptosis, necroptosis, and ferroptosis inhibitors to alleviate the cell death caused by gene overexpression. The research findings demonstrated that the ferroptosis inhibitor could significantly restore cell death caused by gene interference, and the apoptosis inhibitor could also partially restore cell death. Therefore, we speculated that the main reason for the massive cell death after overexpressing FXYD6 was that the cells underwent ferroptosis. In the detection of ferroptosis-related markers and lipid peroxidation, the same conclusion was also drawn; that is, the cells underwent ferroptosis.

Based on the above conclusions, we attempted to speculate on the reasons for this phenomenon. Starting from the function of the gene, FXYD6 has the functions of electron transfer and sodium ion binding of the Na+/K+-ATPase. As one of the core proteins in cell energy metabolism, the Na+/K+-ATPase is closely related to the metabolic reprogramming of cancer cells. To adapt to the need for rapid growth, the expression of the FXYD6 gene would adaptively decrease, thus avoiding the death of cancer cells due to metabolic disorders. In addition, the Na+/K+-ATPase is closely related to the production of intracellular reactive oxygen species (ROS). Excessive expression of FXYD6 would cause the inactivation of the Na+/K+-ATPase to reduce the production of ATP in tumor cells. To adapt to the low-energy mode, cancer cells would generate a large amount of ROS, thus triggering the ferroptosis phenomenon in cancer cells.

Compared with the studies published earlier, our study has several significant differences. For obtaining stable genes, we did not extract the DEGs between OC and normal individuals in a conventional way. Firstly, we divided OC into two subgroups by using the optimal classification algorithm of ML and then extracted the genes with the most significant differences from these two subgroups. Therefore, conventional survival analysis might find that they have no significance for survival. However, for our classification and selection algorithm, these genes are precisely the most crucial ones for the population with different prognoses. The value of conventional differential gene and survival analysis in the prognostic evaluation of OC patients still needs to be improved. For these reasons, we designed this experiment and protocol. Regarding the data used for analysis, we did not start with conventional protein-coding or lncRNA. Through multi-omics integration, we conducted multi-omics exploration from four common dimensions. Methylation data of OC was not included because a large number of literature and our obtained OC methylation data analysis showed that the variation difference of OC methylation data compared with other tumors was very small. Therefore, to make the algorithm robust, we did not incorporate the methylation data into our study. For risk score calculation, starting from the currently conventional algorithms, we generated a large-sample, multi-dimensional model construction and synthesis method by combining these common algorithms. The conclusions drawn from it are better than those of our previous analysis and the algorithms retrieved from the current literature. In the immune feature analysis, we integrated the currently common algorithms used to evaluate immune features. By analyzing more than 1,000 results, we obtained the most comprehensive immunological features, providing a solid basis for practically evaluating the therapeutic effects among molecular subtypes. Besides, in addition to using the validation set to evaluate the effect of molecular classification, we collected samples of OC patients in our hospital. Through IHC and RT-PCR, we systematically analyzed 200 and 6 samples of OC patients who visited our hospital and were pathologically diagnosed, respectively. Through the external cohort, we evaluated the robustness of the conclusions again. *In vitro* cytological experiments, we first determined the role of FXYD6 in the proliferation and migration abilities of OC and speculated on the possibilities of the occurrence of this situation after overexpressing FXYD6. Eventually, we focused our research on ferroptosis. Nevertheless, we acknowledge that there are still some limitations in our study. For instance, the cohorts we utilized varied in terms of size and sequencing platforms; nevertheless, correction methods were employed to mitigate these disparities. Furthermore, additional investigation into the precise mechanism of the tumorigenesis-related activity of the risk score genes is warranted. Additionally, bigger prospective multi-center cohorts are needed to more thoroughly validate the risk score’s clinical relevance.

## Data Availability

Publicly available datasets were analyzed in this study. This data can be found here: GEO (https://www.ncbi.nlm.nih.gov/geo/); GSE26193, GSE49997.
