# Supplementary material for: Identification of prognostic subtypes and the role of FXYD6 in ovarian cancer through multi-omics clustering
Source: Front Immunol. 2025 Mar 18;16:1556715. doi: 10.3389/fimmu.2025.1556715 (PMC11958163; doi:10.3389/fimmu.2025.1556715)

Supplementary Material

# Western Blotting original data

##
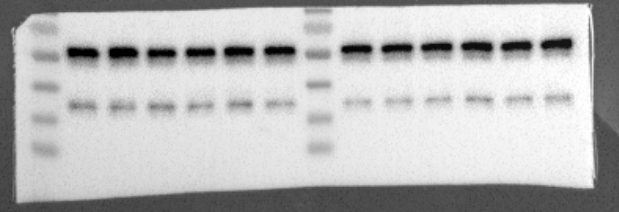

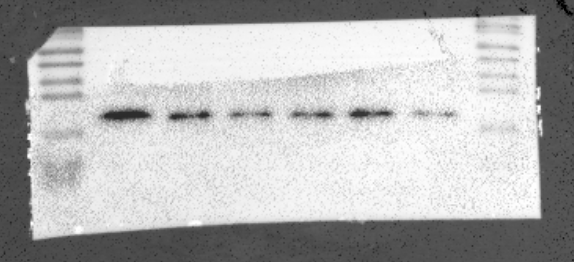
Figure 5C original data

## Figure 5D original data


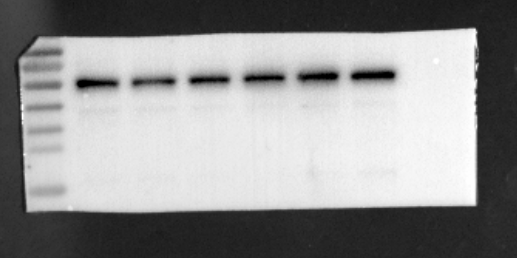

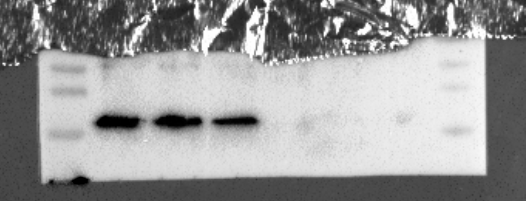


##
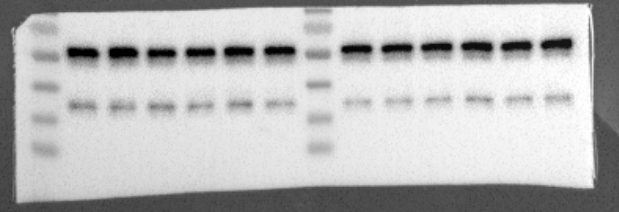

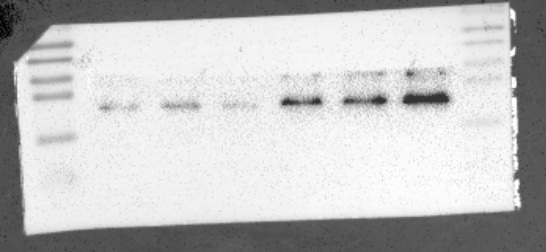
Figure 5F original data

##
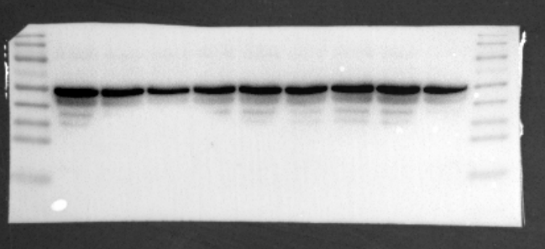
Figure 6C original data


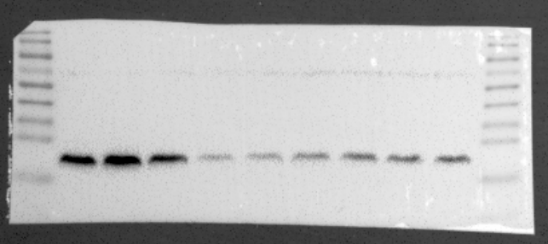

Supplement: Supplementary file 1 [file DataSheet1.docx]
