# Supplementary material for: Identification of prognostic subtypes and the role of FXYD6 in ovarian cancer through multi-omics clustering
Source: Front Immunol. 2025 Mar 18;16:1556715. doi: 10.3389/fimmu.2025.1556715 (PMC11958163; doi:10.3389/fimmu.2025.1556715)
Supplement: Supplementary file 2 [file DataSheet2.docx]

Supplementary Material

## Supplementary Figures


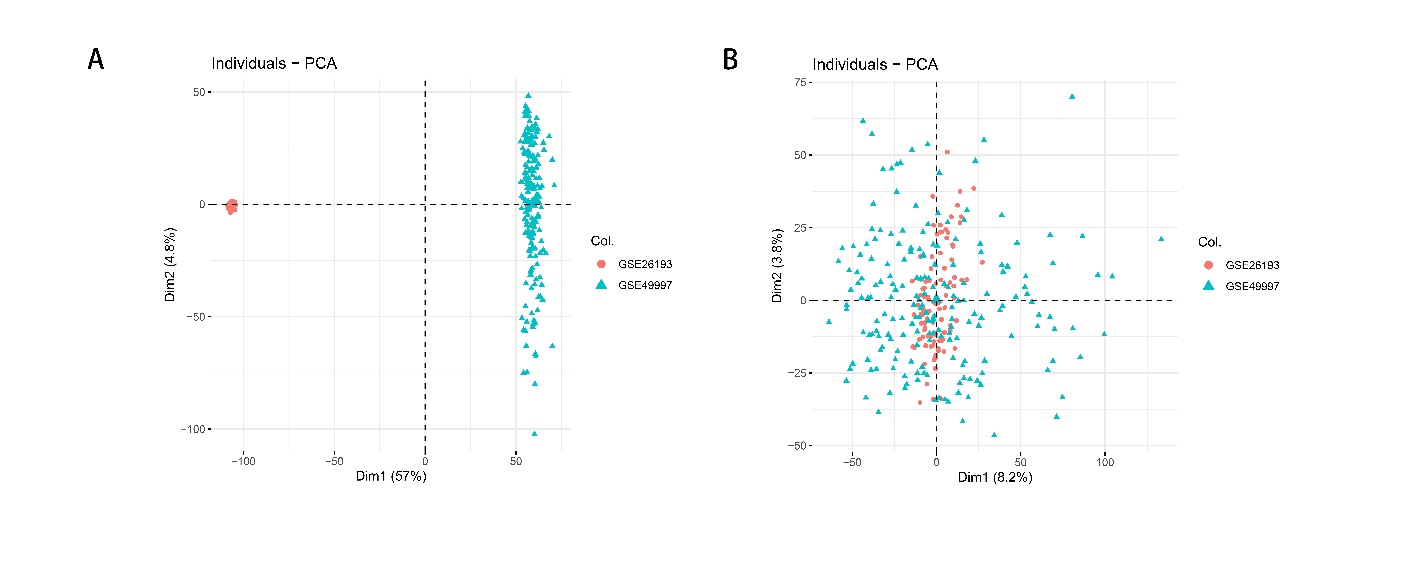


**Supplementary Figure 1.** Distribution of Each Dataset Before and After Batch Correction by PCA Algorithm A: Before batch correction. B: After batch correction.


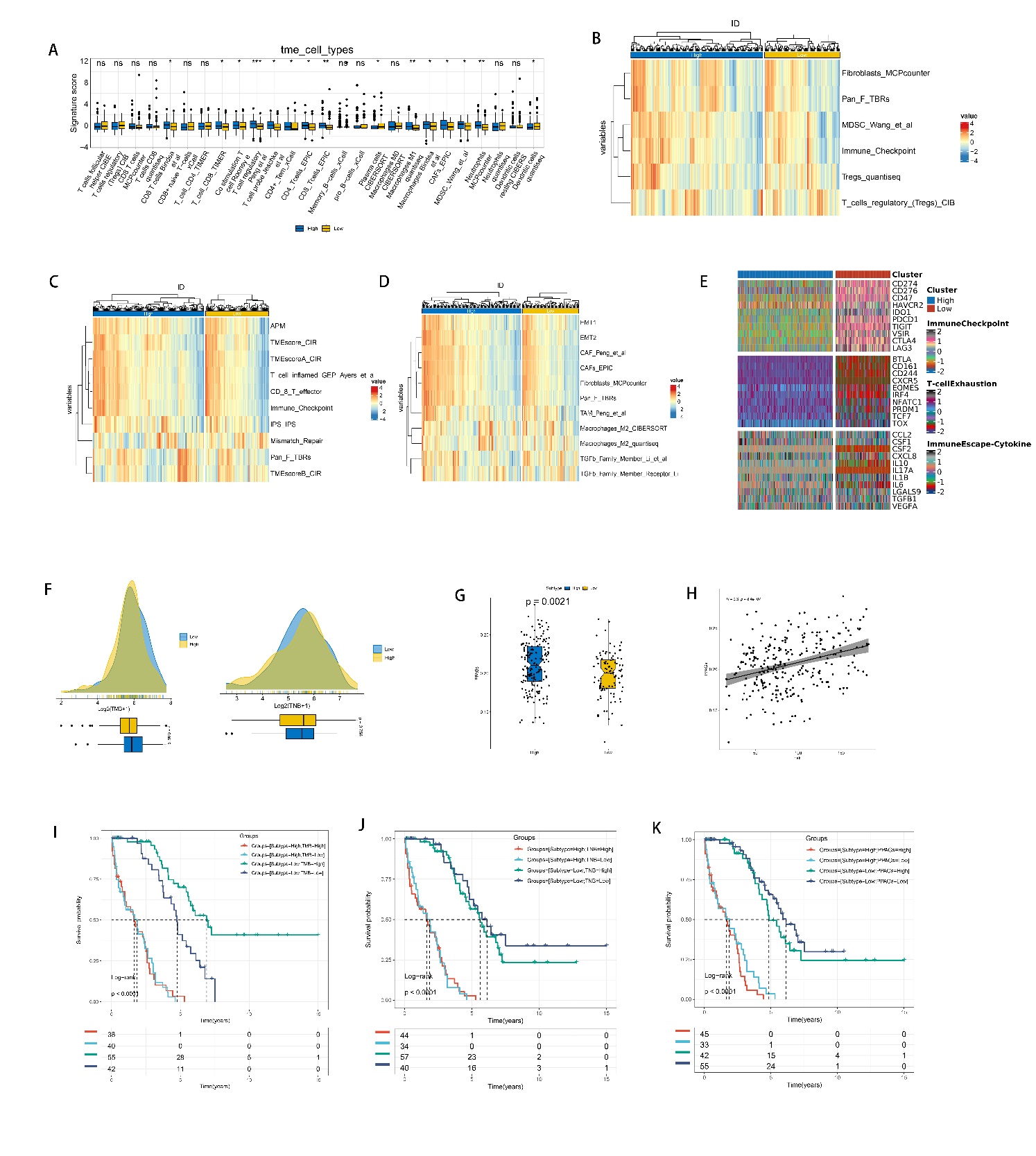


**Supplementary Figure 2.** Risk Scores and Molecular Characteristics of the Immune Microenvironment. A: Distribution of immune cell type characteristics in the tumor microenvironment (TME) among patients with high and low risk scores. B: Distribution of immunosuppressive characteristics among patients with high and low risk scores. C: Distribution of immunotherapy biomarkers among patients with high and low risk scores. D: Distribution of immune exclusion characteristics among patients with high and low risk scores. E: Differences in cytokines associated with immune checkpoints, T cell depletion, and immune escape in high-low risk groups. F: Distribution of tumor mutational burden (TMB) and tumor neoantigen burden (TNB) among patients with high and low risk scores. G: Distribution of programmed death-ligand 1 (PD-L1)-associated genes (PPAGs) among patients with high and low risk scores. H: Relationship between PPAGs and risk scores. I - K: Survival analysis after combining risk scores with TMB, TNB, and PPAGs.


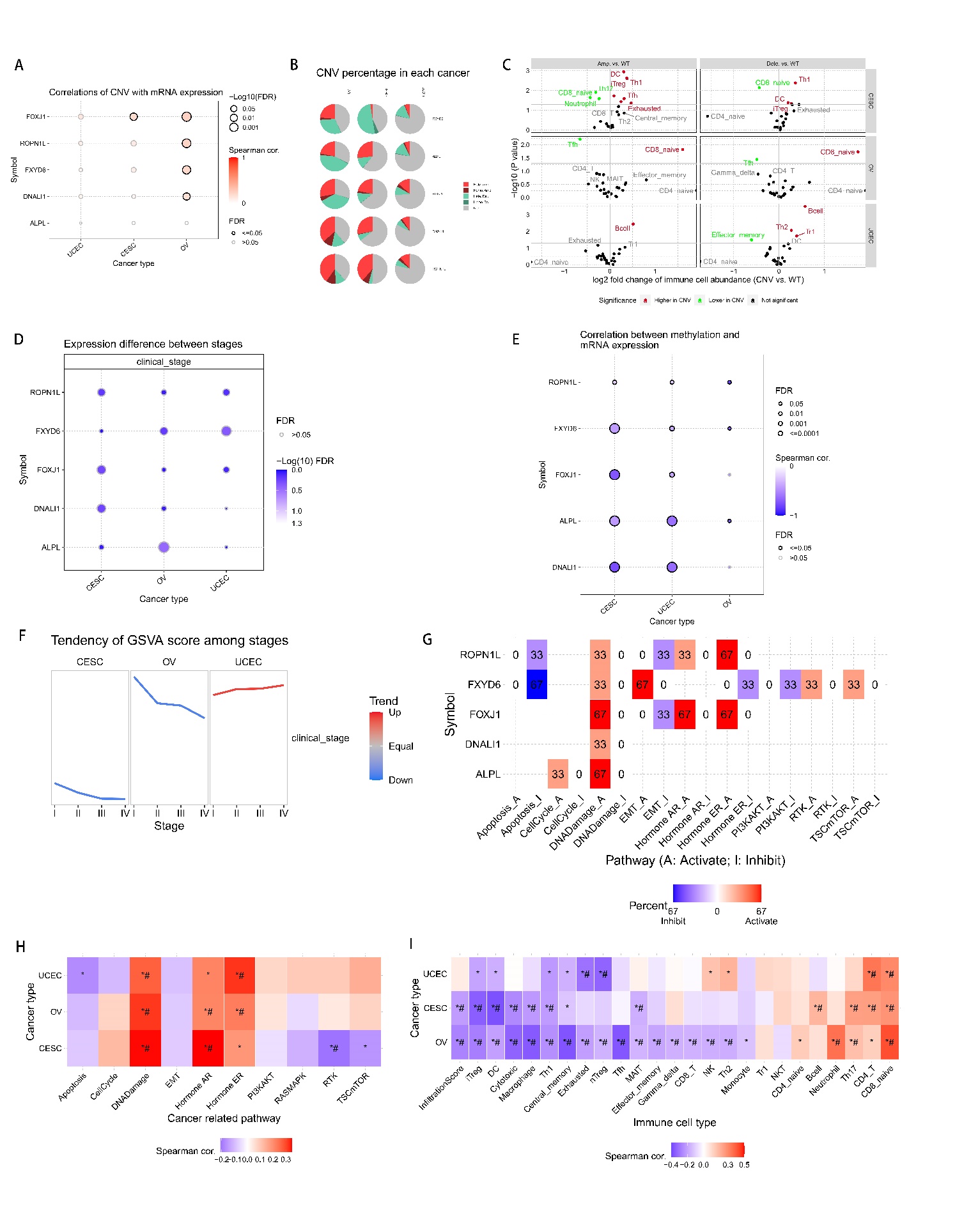


**Supplementary Figure 3.** Correlations of Copy Number Variation, Clinical Stage, Methylation and Immune-related Pathways of Hub Genes**.** A: Correlation between the mRNA expression levels of Hub genes and copy number variation (CNV) in gynecological malignancies. B: Proportion of different types of copy number variation of Hub genes in gynecological malignancies. C: Differences in immune cells between high and low copy number variations of Hub genes in gynecological malignancies. D: Correlation between Hub genes and clinical stage in gynecological malignancies. E: Correlation between the mRNA expression levels of Hub genes and methylation in gynecological malignancies. F: Trend of changes in the GSVA scores of Hub genes and clinical stage in gynecological malignancies. G: Correlation between Hub genes and immune activation and immune suppression in ovarian cancer. H: Correlation between Hub genes and activation status of tumor-related pathways in gynecological malignancies. I: Correlation between Hub genes and types of immune cells in gynecological malignancies.


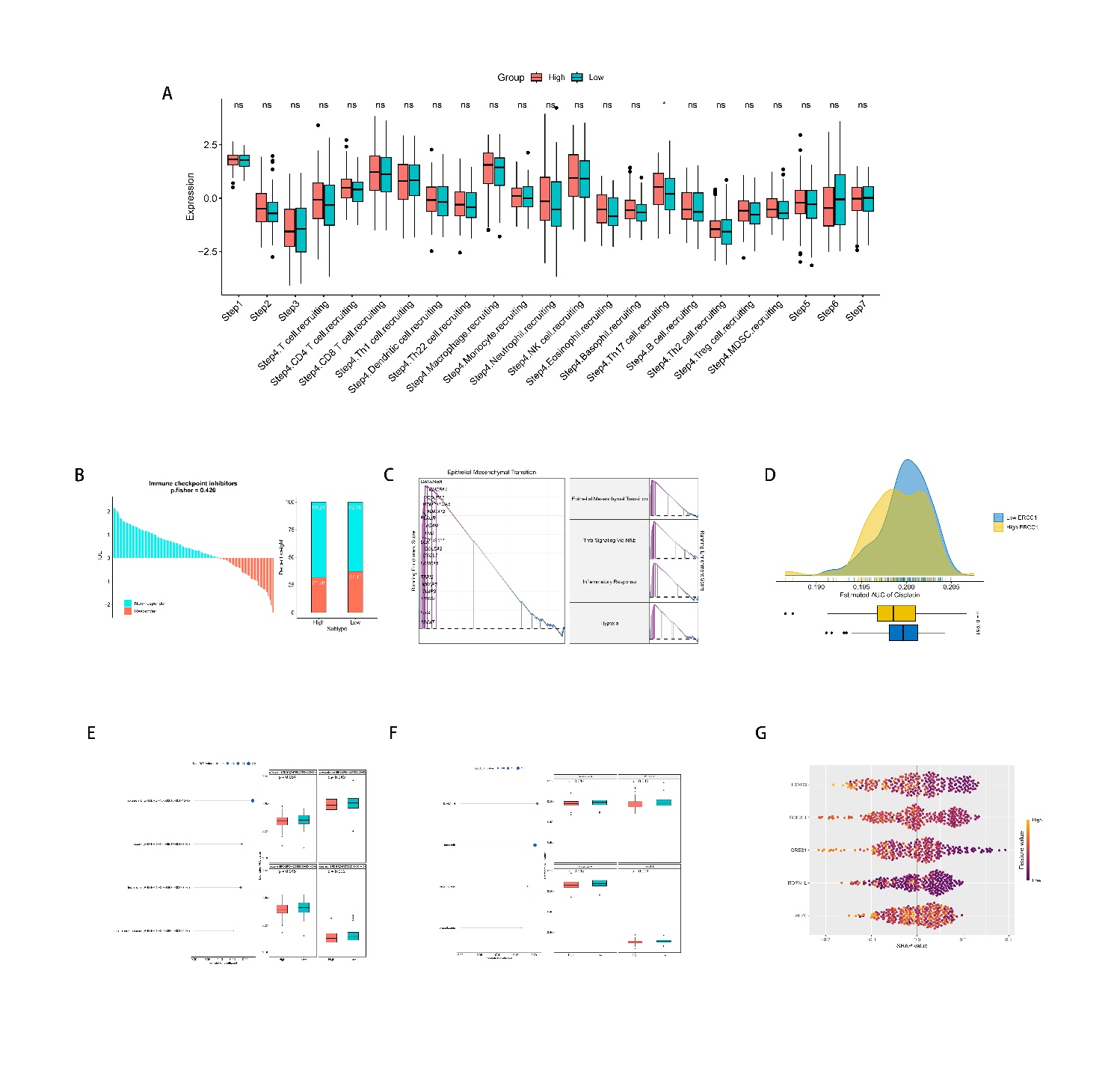


**Supplementary Figure 4.** Potential Drug Screening

A: Differences in the activation levels between high and low risk scores in each step of TIP. B: Prediction of the responses to immunotherapy for high and low risk scores by the TIDE algorithm. C: Pathways significantly activated in the high-risk score group were identified through the GSEA algorithm. D: Pre-prediction of platinum sensitivity to verify the feasibility of the computational algorithm. E - F: Correlation and difference analyses of the drug sensitivity of potential drugs screened from the CTRP and PRISM datasets. G: SHAP analysis evaluated the weight of each gene in the risk model.
